# Matrix Rhythm Therapy as a Novel Clinical Approach in the Rehabilitation of Surgically Treated Distal Radius Fracture: A Single Case Study

**DOI:** 10.7759/cureus.54785

**Published:** 2024-02-23

**Authors:** Pallavi R Bhakaney, Om C Wadhokar, Sakshi Upase

**Affiliations:** 1 Cardiorespiratory Physiotherapy, Dr. D. Y. Patil College of Physiotherapy, Pune, IND; 2 Public Health, Jawaharlal Nehru Medical College, Datta Meghe Institute of Higher Education and Research, Wardha, IND; 3 Physiotherapy, Dr. D. Y. Patil College of Physiotherapy, Pune, IND

**Keywords:** rehabilitation, physical therapy, novel treatment, matrix rhythm therapy, distal radius fracture, case report

## Abstract

Distal radius fractures (DRFs) are prevalent among all hand injuries, commonly due to a fall on an outstretched hand. Not being treated properly can cause many complications such as malunion, non-union, reduced range of motion, and muscle strength. This case report presents a multidisciplinary approach to the physiotherapeutic management of a DRF treated with closed reduction internal fixation using K-wires. The rehabilitation protocol incorporated matrix rhythm therapy (MRT), a novel therapeutic technique, in combination with targeted therapeutic exercises. The study outlines the patient's journey from injury to recovery, detailing the integration of MRT sessions alongside conventional physiotherapy exercises. The comprehensive rehabilitation aimed to enhance pain relief, restore range of motion, and improve functional outcomes. The case highlights the synergistic benefits of incorporating MRT into traditional rehabilitation strategies through assessment, personalized treatment planning, and regular progress evaluations. The outcomes underscore the potential of this combined approach in optimizing the recovery process.

## Introduction

Distal radius fracture (DRF) is one of the most common bone fractures, contributing to nearly one of every five fractures in people aged 65 years or older [1]. The break usually happens due to falling on an outstretched or flexed hand. Signs include pain, swelling, and difficulty moving the wrist or hand. These can vary in severity, from simple fractures that may only require a cast to more complex fractures that may require surgical interventions. Complications of a DRF can arise during the delayed healing curative process or after treatment. These injuries can result in a higher incidence of long-term functional limitations, pain, and physical deformities [2]. Some potential complications include malunion, non-union, nerve and blood vessel damage, complex regional pain syndrome, stiffness, reduced range of motion, infection, chronic pain, etc. Physiotherapy has been an effective method to restore full joint movement and improve functional ability [3]. Additionally, massage therapy is widely considered a soft tissue manipulation technique that enhances blood circulation. A newly discovered vibro-massage method, called matrix rhythm therapy (MRT), is the latest advancement used in the field of physiotherapy. The MRT originates from research Dr. Randoll conducted at Erlangen University in the 1990s. It is a therapeutic device designed to stimulate and restore specific physiological vibrations in skeletal muscles and the nervous system [4]. This therapy enhances the natural rhythm of muscles and indirectly regulates associated processes. MRT is said to regulate as well as balance the normal physiological rhythm due to resynchronization. Due to the inflammatory process, restriction to the flow of oxygen causes restricted movement. Oscillations ranging from 8 to 12 Hz result in improved microcirculation, which, in turn, causes muscle relaxation. MRT has numerous effects on body systems ranging from physiological and chemical to physical. This method has a broad range of applications [5]. This case study demonstrates the efficiency of MRT in improving the targeted goal in a case of postoperative DRF.

## Case presentation

A 61-year-old housewife reported a history of a fall in her house due to the slippery floor and experienced instant sharp pain and swelling on her right wrist. On admission to the hospital, a preliminary assessment was done, which included a series of investigations, including X-ray imaging and laboratory investigations like kidney function tests, calcium levels, vitamin D levels, etc. These revealed low levels of calcium and vitamin D. An X-ray of the right wrist showed a fracture of the distal radius. No relevant medical or pharmacological history was reported by the patient. Surgical intervention was planned, which included a closed reduction internal fixation with K-wire. The patient was discharged from the hospital on the third day and was called for follow-up regularly. Twenty days post-surgical treatment, suture removal was done, and antibiotics were prescribed. On the 2nd of October, the implant embedded within the patient was removed. The patient visited Dr. D. Y. Patil College of Physiotherapy Outpatient Department (OPD) with complaints of pain and stiffness in her right wrist around 28 days after the last follow-up of implant removal. On admission to the Physiotherapy OPD, her primary complaints included pain and inability to move her right wrist, associated with aggravating right lateral thumb pain. She gave a detailed history of a series of medical conditions, which include diabetes mellitus for 18-20 years (on medications), a left frozen shoulder three years ago, and chronic bronchitis for five years. She also mentioned the complaint of experiencing breathlessness after walking on an inclined surface.

During the general examination, the patient was oriented to time, place, and person. Initial assessment included postural assessment, which revealed the right shoulder was slightly elevated. Palpation of the right forearm revealed evident muscle wasting of the brachioradialis. There was a rise in the local body warmth; however, when checked for any sensory deficits, no positive findings were revealed. Besides this, weakness of finger abductors and adductors was seen, along with tightness in the brachioradialis muscle. The range of motion assessment was done before the commencement of the treatment, and after four weeks of the physiotherapy treatment, the range of motion of the right shoulder and left upper limb is full and functional. The pre- and post-range of motion and strength assessment is mentioned in Table 1.

**Table 1:** The pre-treatment (day 1) and post-treatment range of motion (week 4) and grades of manual muscle testing of wrist and elbow

Joint	Movement	Range of motion pre-treatment	Range of motion post-treatment	Muscles	Grade pre-treatment	Grade post-treatment
Right wrist	Flexion	0-19°	0-44°	Flexors	2/5	4/5
	Extension	0-25°	0-46°	Extensors	2/5	4/5
	Radial deviation	0-10°	0-15°	Ulnar deviators	2/5	4/5
	Ulnar deviation	0-15°	0-23°	Radial deviators	2/5	4/5
	Supination	0-45°	0-75°	Supinator	2/5	4/5
	Pronation	0-65°	0-80°	Pronators	2/5	4/5
Right elbow	Flexion	0-130°	0-135°	Flexors	4/5	5/5
	Extension	130-0°	135-0°	Extensors	4/5	5/5

**Figure 1:**
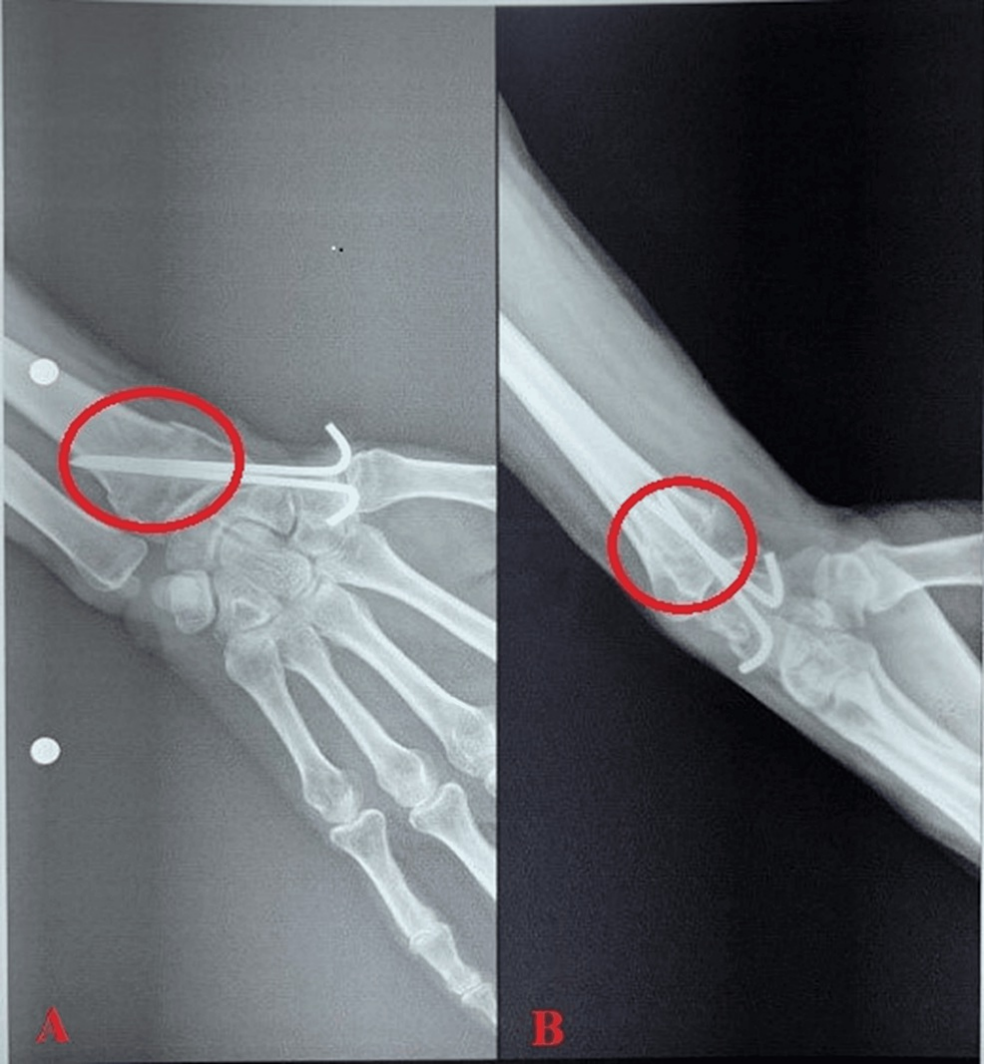
(A) AP view of the right wrist. (B) Lateral view of the postoperative distal end radius fracture managed with K-wire fixation. AP, anteroposterior

The below-mentioned X-ray shows postoperative distal end radius fracture managed with K-wire fixation (Figures 1A-1B).

The goal of rehabilitation was directed toward managing pain, regaining functional range and strength, and improving the quality of life of the patient. With pertinent rehabilitation, the achievable outcomes were measured on outcome measures assessing overall functioning at the end of four weeks. The short-term goals were to manage pain, reduce swelling, improve the range of motion at the wrist, elbow, and phalanges, improve muscle strength and grip strength, followed by achieving the independence to perform the activities of daily living. A tailored treatment protocol was given to the patient considering the abovementioned goals to be achieved; the detailed treatment protocol is mentioned in Table 2, with the dosage and rationale for each intervention.

**Table 2:** Rehabilitation protocol

Intervention	Dosage	Rationale
Cryotherapy	10 minutes thrice a day over the anterior aspect of the wrist	Application of ice reduces the nerve conduction velocity, causing damping of pain signals to a higher center
Active range of motion exercise (wrist musculature)	10 repetitions of each movement twice daily	To maintain muscle pliability, avoid muscle atrophy, and prevent muscle wasting
Maitland mobilization (wrist, metacarpophalangeal, interphalangeal joints, and proximal radio-ulnar joint)	Grades 2 and 3 mobilizations, 10 oscillations, three sets	To break capsular adhesions, improve joint play, reduce pain, and augment the range of motion at the wrist
Matrix rhythm therapy (MRT)	30 minutes over the anterior aspect of the wrist and forearm	The oscillation by MRT causes the re-establishment of the disturbed cellular frequency, resolves stiffness, augments tissue healing, and improves the range of motion
Ischemic compression technique	90 seconds, three sets	Causing a local increase in circulation, promoting tissue healing
Muscle energy techniques (MET)	20% of maximum, seven seconds hold, 10 repetitions	Activation of the Golgi tendon organ induces relaxation of the antagonist, accentuating the strengthening of the agonist
Gripping exercises	10 repetitions thrice a day using a resistance band/smiley ball	Strengthening lumbricals, palmar, and dorsal interossei
Dexterity exercise	10 repetitions thrice a day	Improving overall hand functionality

**Figure 2:**
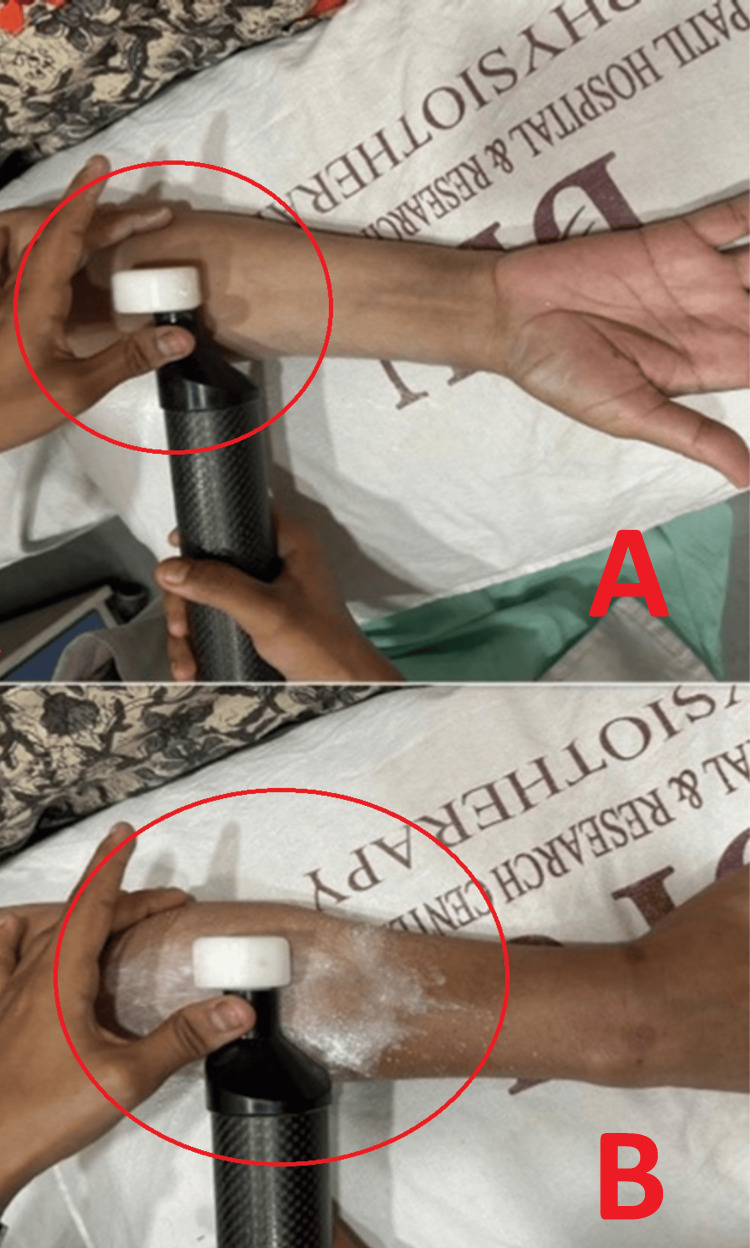
(A) Matrix rhythm therapy applied with the treatment head on the anterior compartment of the forearm. (B) Matrix rhythm therapy applied with the treatment head on the posterior compartment of the forearm.

The MRT was started in the rehabilitation protocol on day 2. The patient was instructed not to consume tea, coffee, or alcohol since the morning till the time of the treatment, which can hamper the blood supply. The MRT was applied to the interosseous membrane for about 30 minutes, with a frequency of 8-12 Hz, parallel to the fibers of muscle groups, using solid lubricant to reduce friction over the skin (Figure 2). The treatment was given at a frequency of three times per week.

The effectiveness of the rehabilitation protocol was measured using the following outcome measures (Table 3).

**Table 3:** Effectiveness of the treatment on the outcome measures

Outcome measures	Day 1 (pre-treatment)	Week 4 (post-treatment)
Numerical Pain Rating Scale (NPRS)	8/10 on activity	3/10 on activity
Disability of Arm, Shoulder, and Hand (DASH)	76	45
Patient-Rated Wrist Evaluation (PRWE)	85	40

## Discussion

The case report focuses on the documentation of analyzing the combined impact of MRT and therapeutic exercises in managing DRF. By combining traditional surgical interventions with advanced physiotherapeutic techniques like MRT, clinicians can optimize the healing process, improve patient outcomes, and enhance overall quality of life [6]. The successful integration of these modalities emphasizes the potential for their broader application in similar cases, encouraging further exploration and research in orthopedic physiotherapy. This combined approach influences clinical decision-making, emphasizing its potential to improve outcomes and patient satisfaction [7]. The objective in surgically treating DRF is to achieve the proper anatomical reconstruction of the joint surface, ensure stable fixation, and promote effective wrist and forearm motion [8]. Blomstrand et al. assessed the level of pain, the function of the hand, and activity performance in surgically treated DRF patients. They used the conventional methods of treatment, which included a range of motion exercises and overall hand functional exercises, and continuously focused on the strengthening exercises; their conclusion, however, is consistent with the findings of this case study, where all the factors were improved [9]. Moreover, novel methods of treatment in the field of physiotherapy such as Factor et al.’s pilot study on the application of pulsed electromagnetic field (PEMF) to 41 patients with DRF. The outcomes were measured on pain, range of motion, grip strength, and quality of life, which were seen to be improved [10]. Low-level laser therapy is also effective in relieving pain and improving the healing of closed bone fractures in the human wrist and hand [11]. Virtual reality is emerging in rehabilitation, as documented by Kulkarni et al., where virtual reality-based rehabilitative exercises were proposed to be administered using a head-mounted device, Oculus Quest. Kulkarni et al. hypothesized that virtual reality in DRF patients has proven to be effective in improving the condition of the patient and has also been seen as a non-exhaustive patient rehabilitation [12].

## Conclusions

The case report highlights the promising outcomes achieved through a comprehensive treatment approach for DRFs and the integration of MRT alongside targeted therapeutic exercises. The synergistic effects of these interventions have significantly contributed to the patient's recovery and rehabilitation process. MRT, a unique and innovative physiotherapeutic modality, was pivotal in reducing pain, improving tissue dynamics, and enhancing overall healing. When coupled with tailored therapeutic exercises, the therapy helped restore range of motion, muscle strength, and functional abilities, leading to a more holistic recovery experience for the patient. As we move forward, healthcare professionals must continue exploring innovative and evidence-based therapies, such as MRT, to further refine rehabilitation protocols and enhance the recovery experiences of patients with DRFs. This case serves as a valuable reference, demonstrating the positive impact of a well-coordinated, multidisciplinary approach in achieving favorable outcomes and improving the lives of individuals recovering from complex fractures.
